# Comparison of venous and calculated blood gas values to arterial values in critically ill patients

**DOI:** 10.1111/aas.14555

**Published:** 2024-11-24

**Authors:** Mads Lumholdt, Jo Bønding Andreasen, Kjeld Damgaard, Erika Frischknecht Christensen, Peter Derek Christian Leutscher, Bodil Steen Rasmussen

**Affiliations:** ^1^ Department of Anaesthesiology and Intensive Care North Denmark Regional Hospital Hjørring Denmark; ^2^ Department of Anaesthesiology and Intensive Care Aalborg University Hospital Aalborg Denmark; ^3^ Centre for Clinical Research North Denmark Regional Hospital Hjørring Denmark; ^4^ Department of Clinical Medicine Aalborg University Aalborg Denmark; ^5^ Centre for Prehospital and Emergency Research Aalborg University Hospital Aalborg Denmark

**Keywords:** acid–base, arterial blood gas, calculated blood gas, critical care, ICU, venous blood gas, v‐TAC

## Abstract

**Background:**

Arterial blood gas (ABG) values are important in the assessment of critically ill patients. However, arterial puncture may be challenging to perform in these patients. The venous‐to‐arterial conversion method (v‐TAC) is used to convert venous blood gas values to calculated values meant to resemble arterial values. Calculated pH and partial pressure of carbon dioxide (PCO_2_) values have shown good agreement with arterial values in stable patients, but performance of the method in patients with severe acid–base disturbances is unknown. We aim to evaluate venous and calculated blood gas value congruency with arterial values in critically ill patients.

**Methods:**

Critically ill adult patients with abnormal arterial pH (<7.35 or >7.45) admitted to an intensive care unit were included in this study. Patients were divided in groups based on arterial pH (alkalemia, moderate acidaemia and severe acidaemia). ABG samples were obtained from arterial catheters and venous samples simultaneously from peripheral or central venous catheters. Venous blood gas values were converted to calculated values using the v‐TAC software. Both venous and calculated values of pH, PCO_2_ and partial pressure of oxygen (PO_2_) were compared to arterial values in scatterplots and using the Spearman's Rank Correlation.

**Results:**

Arterial and venous sample pairs were obtained from 28 patients, and 24 venous values were converted using the v‐TAC method. Scatterplots showed similar congruency of venous and calculated pH and PCO_2_ values to arterial values. However, one patient in the severe acidaemia group had arterial pH 7.07 and venous pH 7.04, but calculated pH 7.42. Spearman's Rank Correlation rho showed no correlation of venous and calculated PO_2_ values compared to arterial values (*p* = .989 and *p* = .361).

**Conclusion:**

Venous and calculated pH and PCO_2_ values showed similar congruency with arterial values in patients with alkalemia and moderate acidaemia, while the method was unreliable in a patient suspected of severe metabolic acidaemia.

**Editorial Comment**

This study compared how the commercially available venous‐to‐arterial conversion method performed in estimating the results of arterial blood measurements from a venous blood sample and a measurement of oxygen saturation in a cohort of critically ill patients with abnormal pH. There was a good correlation between pH and partial pressure of carbon dioxide (PCO_2_) measurements in venous samples and both were close to measured values in the corresponding arterial sample, although the predicted value was inaccurate with severe acidemia. Most of the saturation measurements were above the calibration range of the software and therefore defaulted to ‘high.’ There remains uncertainty concerning how precise and accurate estimates of arterial blood gas PCO_2_ and pH values with this method would be in extreme cases.

## 1 INTRODUCTION

Arterial blood gas (ABG) values are important in the assessment and monitoring of oxygenation, ventilation and acid–base status in critically ill patients.^1^, ^2^ However, arterial puncture or catheterisation may be challenging to perform in patients with low blood pressure, poorly palpable or weak pulse.^3^, ^4^ McKeever et al. found that arterial puncture was successful after one attempt in 69.2% of patients admitted with acute exacerbation of chronic obstructive pulmonary disease (AECOPD), while venous puncture to obtain venous blood gas samples was successful after one attempt in 90.2%.^5^ The appealing accessibility of venous puncture has led to several studies comparing arterial and venous blood gas values. A systematic review and meta‐analysis from 2014, with 18 studies comparing arterial and peripheral venous values, found minor discrepancy between arterial and venous pH, while venous partial pressure of carbon dioxide (PCO_2_) and partial pressure of oxygen (PO_2_) were inaccurate compared to arterial values.^6^ Of note, the discrepancy between venous and arterial PCO_2_ and PO_2_ was more pronounced in hemodynamically unstable patients and those with congestive cardiac failure.^6^ More recent studies have shown similar results, both for peripheral and central vein samples, in critically ill patients admitted to an intensive care unit (ICU),^7^, ^8^ and specifically in mechanically ventilated patients admitted to an emergency department (ED).^9^, ^10^ Various mathematical models have been developed to predict arterial values from peripheral and central venous samples.^11^, ^12^, ^13^, ^14^ These methods vary from simple correction factors, derived from linear regression multiplied with venous values,^12^ to advanced ‘arterialisation’ by artificial neural networks.^13^ One method, the venous‐to‐arterial conversion method (v‐TAC, OBI Medical/Roche, Denmark/Switzerland), has gained international attention.^14^, ^15^, ^16^, ^17^, ^18^ The principles of the method have been described by its inventors.^14^ In short, it is based on five steps; (A) an anaerobic venous sample is drawn and peripheral oxygen saturation (SpO_2_) is measured by pulse oximetry, (B) venous pH, PCO_2_, PO_2_, SO_2_, haemoglobin (hb), methaemoglobin, carboxyhaemoglobin and electrolytes are used to calculate the venous total carbon dioxide (tCO_2,v_) and oxygen (tO_2,v_) concentration, base excess (BE), concentration of 2,3‐diphospoglycerate (DPG) and total non‐bicarbonate buffer base (tNBB), (C) the arterial total carbon dioxide (tCO_2,a_) and oxygen (tO_2,a_) concentration are calculated using the respiratory quotient (RQ) in the formula ΔCO_2_ = RQ × ΔO_2_, (D) tCO_2,a_, tO_2,a_, hb, BE, tNNB and DPG are then used to calculate arterial pH, PCO_2_, PO_2_ and SO_2_, (E) the calculated arterial SO_2_ is then compared to the SpO_2_ measured by peripheral pulse oximetry. The difference between the two is used to repeat steps (C)–(E), whereupon the calculated pH, PCO_2_ and PO_2_ should be equal to measured arterial values.^14^

The v‐TAC method has been evaluated in several studies with hemodynamically stable patients without severe acid–base disturbances, and the agreement between arterial and calculated pH and PCO_2_ has been high, although calculated PO_2_ is overestimated if arterial PO_2_ values were above 8–12 kPa.^14^, ^15^, ^16^, ^17^, ^18^, ^19^, ^20^, ^21^ In one study, adult patients with respiratory failure were admitted to either an ICU (*n* = 32) or to a pulmonary medical ward (*n* = 59), and while the calculated pH and PCO_2_ values approximated arterial values well, calculated PO_2_ values were less precise.^21^

The v‐TAC method has not been evaluated in critically ill patients with pronounced organ dysfunctions. The aim of this study was to evaluate venous and calculated blood gas value congruency with arterial values in critically ill patients admitted to the ICU with severe acid–base disturbance defined as an abnormal arterial pH at baseline.

## 2 METHODS

### 2.1 Setting

This comparative study was conducted at the ICU at North Denmark Regional Hospital, a secondary care hospital in the North Denmark Region, Denmark. This hospital offers 24‐h acute care facilities, and the 7‐bed ICU receives both surgical and medical patients.

### 2.2 Population

Critically ill adult patients admitted to the ICU between December 2017 and December 2018 were considered for inclusion in this study. Patients were included if arterial catheters were required for other reasons and at least one venous access, central and/or peripheral, was available. Sample pairs were collected as convenience samples, and physicians were instructed not to let sampling interfere with lifesaving treatment. Samples were only collected if the arterial pH value was outside of normal reference interval, pH <7.35 or >7.45.^22^

### 2.3 Patient data

Information on patient characteristics, vasoactive and/or inotropic therapy, the Simplified Acute Physiology Score 3 (SAPS‐3) and reason for admission score was registered upon every sample pair collection. In the recalculation of the SAPS‐3 score, in patients with multiple samples collected within few hours, only the patients' vital parameters were updated, as measurement of blood bilirubin, creatinine, leukocytes and platelets was only performed upon admission and on daily basis.

### 2.4 Sample collection and analysis

Guidelines on sample collection were formulated prior to study commencement and ICU staff were familiar with the procedures. Arterial and venous sample pairs were obtained simultaneously by the attending physician and ICU nurse. Multiple sample pairs were collected from each patient. The arterial samples were obtained in accordance with regional standard operating procedure for arterial sampling from intra‐arterial blood pressure monitoring systems connected to arterial catheters.^23^ Venous sampling from peripheral venous catheters was conducted as follows; infusions were halted for 5 min, and 5 mL of blood was aspirated. If venous blood did not flow spontaneously during sampling, a venous stasis torniquet was briefly applied. Continuous infusion considered lifesaving (e.g., noradrenaline) overruled sample collection; thus, in these circumstances, another venous access was used, or sampling abandoned. Venous sampling from central venous catheters were conducted by halting infusions considered safe to pause for 5 min (e.g., antibiotics or crystalloids) and aspirating 5 mL of blood prior to venous sampling. All samples were collected in heparinised *safe*PICO blood gas syringes (Radiometer, Denmark) and analysed immediately using a ABL800 Flex blood gas analyser (Radiometer, Denmark) localised at the ICU. A standard patient body temperature of 37.0°C was used in the blood gas analyser for all patients. The venous pH, PCO_2_ and PO_2_ values were converted to calculated values using the commercially available v‐TAC software (Obimedical/Roche, Denmark/Switzerland) installed on the blood gas analyser. Due to the overestimation of PO_2_ at high SO_2_, the distributors of the v‐TAC method have introduced an upper limit of the reported PO_2_ of >10 kPa. Similarly, the method did not convert samples if SpO_2_ was <75%. The basis for these limitations is unknown.

### 2.5 Ethics statement

This study did not require approval from the North Denmark Ethical Committee according to the Danish law on ethics (acceptance letter: 7 June 2017). The study was approved by the Danish Data Protection Agency (J. 2008‐58‐0028).

### 2.6 Statistical methods

Numerical data were not expected to be normally distributed; thus, non‐parametric statistics were used. Continuous numeric variables are presented as median and interquartile range (IQR) and categorical nominal variables are presented as counts. Arterial values were compared to both venous and calculated values in scatterplots and Spearman's Rank Correlation was calculated for each comparison. To detect differences between arterial, venous and calculated value pairs related to various levels of acid–base abnormality, the pairs were grouped in clusters based on the arterial pH values as follows: severe acidaemia pH ≤7.20, moderate acidaemia pH >7.20 and <7.35 and alkalaemia pH >7.45. In patients with repeated sampling, the pair with the most aberrant arterial pH was identified and used for further analysis. Outlier values were assessed using the IQR rule.^24^ All statistical calculations were conducted using R‐Studio (http://www.r-studio.com, version 1.4.1717).

## 3 RESULTS

### 3.1 Patient characteristics

Table 1 shows the patient characteristics and data registered at sample obtainment. A total of 64 sample pairs were obtained from 28 patients. For each patient, the pair with the most aberrant arterial pH was selected, leaving 28 arterial and venous sample pairs.

**TABLE 1:** Patient characteristics when samples were obtained.

*N*	28
Age (years), median (IQR)	69 (60.5–75.0)
Sex, *N* female/male	11/17
SAPS‐3, median (IQR)	66 (54–82)
Vasopressor therapy, *N*	7
Inotropic therapy, *N*	1
Reason for admission, *N*	
Sepsis, abdominal focus	3
Sepsis, unknown focus	3
AECOPD	3
Sepsis, pulmonary focus	2
Sepsis, urological focus	2
Cardiac arrest	2
Ileus	2
Diabetic ketoacidosis	2
Hypokalaemia	2
ARDS	1
Metabolic acidosis (drug)	1
Congestive heart failure	1
Pneumonia	1
Pancreatitis	1
Kidney failure	1
Unregistered	1

*Note*: Variables were collected when samples were obtained. The Simplified Acute Physiology Score 3 (SAPS‐3) was calculated using most recent routine blood sample values (<24 h old). Reasons for admission were in some cases only the preliminary diagnosis.

Abbreviations: AECOPD, acute exacerbation of chronic obstructive pulmonary disease; ARDS, acute respiratory distress syndrom; IQR, interquartile range.

### 3.2 Limitations of the v‐TAC method

The v‐TAC method was unable to convert four venous samples from patients with severe acidaemia to calculated values; thus, the number of arterial‐calculated value pairs was 24. This was due to inability to convert venous samples with a pH value below 6.8 (one sample), venous hb was not measured (one sample) and peripheral saturation measured by pulse oximetry (SpO_2_) was below 75% (two samples). Thirteen calculated PO_2_ values were above 10 kPa and marked with a ‘>10 kPa’ label as a limitation of the v‐TAC software.

### 3.3 Blood gas samples

Table 2 shows the median (IQR) of arterial, venous and calculated blood gas values categorised by type and severity of acid–base disturbances. Based on arterial pH, 7 patients had severe acidaemia, 16 had moderate acidaemia and 5 had alkalemia. Among the 24 patients, the v‐TAC method was able to calculate blood gas values in 3 with severe acidaemia, 16 with moderate acidaemia and 5 with alkalemia. In total, the median (IQR) could not be calculated for 13 values of calculated PO_2_, as these were marked with the ‘>10 kPa’ label; thus, the median (IQR) of calculated PO_2_ in Table 2 was only based on 11 patients; 1 with severe acidaemia, 8 with moderate acidaemia and 2 with alkalemia.

**TABLE 2:** Median (interquartile range [IQR]) of arterial, venous and calculated blood gas values.

	*N* = 28	Arterial blood gas	Venous blood gas	Calculated values
pH, median (IQR)				
Severe acidaemia	7	7.07 (7.06–7.13)	7.04 (7.03–7.11)	7.18 (7.12–7.30)^a^
Moderate acidaemia	16	7.26 (7.23–7.33)	7.25 (7.22–7.28)	7.26 (7.23–7.32)^a^
Alkalemia	5	7.50 (7.47–7.57)	7.49 (7.47–7.50)	7.50 (7.48–7.56)^a^
PCO_2_, median (IQR)				
Severe acidaemia	7	7.8 (5.0–8.9)	8.2 (5.5–9.6)	8.0 (4.5–9.0)^a^
Moderate acidaemia	16	5.5 (5.1–6.9)	6.1 (5.3–8.5)	5.5 (4.8–7.3)^a^
Alkalemia	5	5.1 (3.7–6.5)	5.0 (4.5–6.7)	4.9 (3.7–6.1)^a^
PO_2_, median (IQR)				
Severe acidaemia	7	11.5 (11.4–15.2)	7.8 (7.4–8.1)	9.6^b^
Moderate acidaemia	16	9.8 (9.0–11.4)	6.2 (5.2–6.7)	8.9 (8.8–9.1)^b^
Alkalemia	5	11.4 (9.8–11.5)	5.1 (3.9–6.7)	7.7 (7.7–7.8)^b^

*Note*: Blood gas values are organised by type and severity of acid–base disturbance by arterial pH; alkalemia >7.45, moderate acidaemia <7.35 and >7.20 and severe acidaemia ≤7.20.

Abbreviations: IQR, interquartile range; PO_2_, partial pressure of oxygen; PCO_2_, partial pressure of carbon dioxide; v‐TAC, venous‐to‐arterial conversion method.

^a^ The v‐TAC method was unable to convert 4 of the 28 venous samples to calculated values. The numbers of calculated values in the table are: severe acidaemia *N* = 3, moderate acidaemia *N* = 16 and alkalemia *N* = 5.

^b^ Additionally, 13 of the 24 calculated PO_2_ values were marked with the label ‘>10 kPa.’ The numbers of numeric calculated PO_2_ values in the table are: severe acidaemia *N* = 1, moderate acidaemia *N* = 8 and alkalemia *N* = 2.

### 3.4 Correlation between sample pairs

**FIGURE 1:**
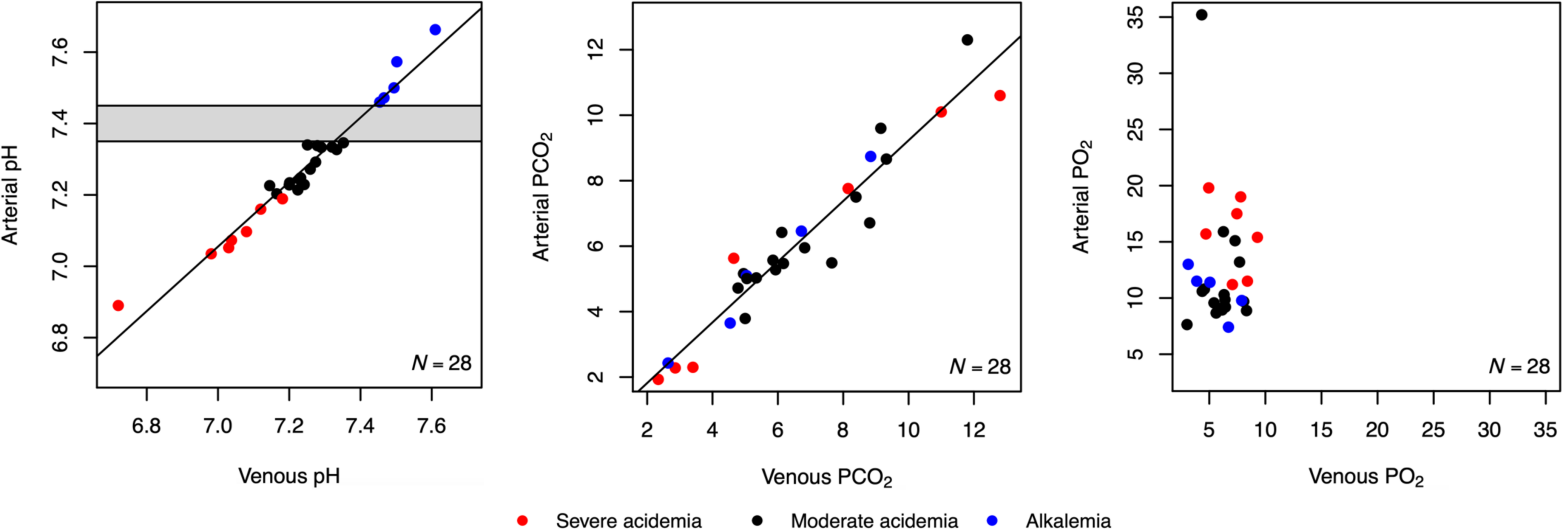
Scatterplots of arterial and venous pH, partial pressure of carbon dioxide (PCO_2_) and partial pressure of oxygen (PO_2_) values. Each point represents one paired venous and arterial value. Points are organised by colour code based on arterial pH; alkalemia >7.45 (blue), moderate acidaemia <7.35 and >7.20 (black) and severe acidaemia ≤7.20 (red).

**FIGURE 2:**
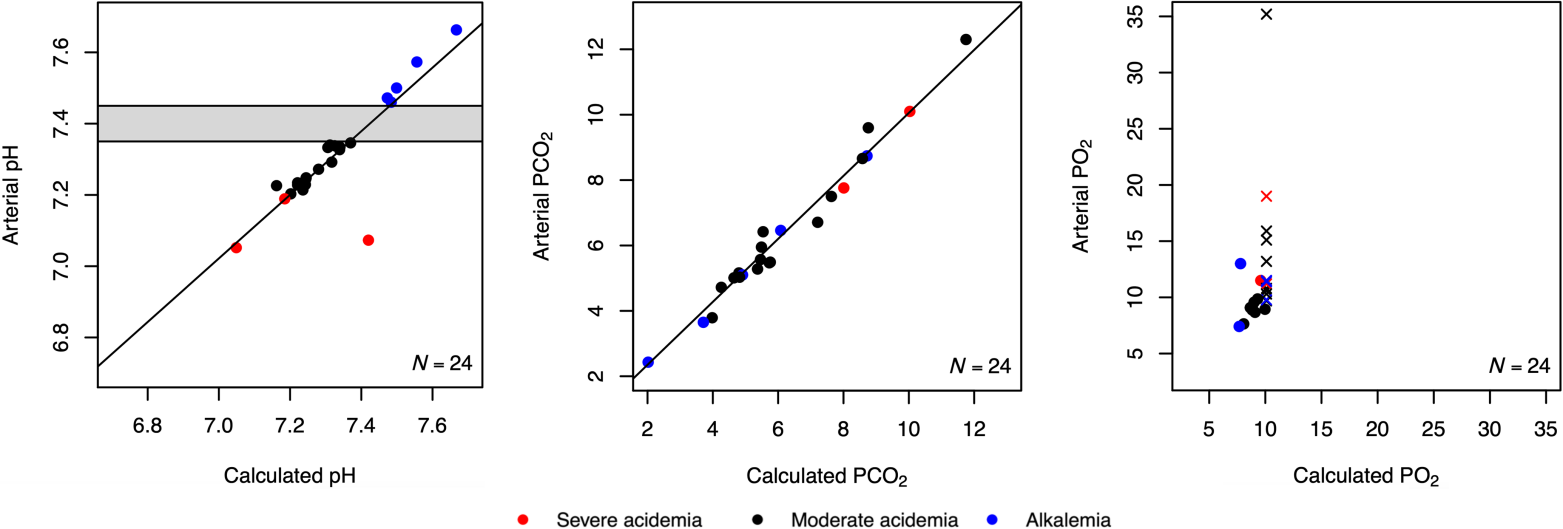
Scatterplots of arterial and calculated pH, partial pressure of carbon dioxide (PCO_2_) and partial pressure of oxygen (PO_2_) values. Venous values were converted to calculated values using the venous‐to‐arterial conversion method (v‐TAC) software. Each point represents one paired calculated and arterial value. Points are organised by colour code based on arterial pH; alkalemia >7.45 (blue), moderate acidaemia <7.35 and >7.20 (black) and severe acidaemia ≤7.20 (red). Crosses in PO_2_ plot represents calculated PO_2_‐values with the ‘>10 kPa’ label applicated by the v‐TAC software.

Figure 1 shows scatterplots between arterial and venous pH, PCO_2_ and PO_2_ values. The Spearman's Rank Correlation rho between arterial and venous pH, PCO_2_ and PO_2_ values from all patients with acidaemia and alkalemia were 0.96 (*p* < .001), 0.96 (*p* < .001) and −0.01 (*p* = .989), respectively. Figure 2 shows scatterplots between arterial and calculated pH, PCO_2_ and PO_2_ values. The correlation rho between arterial and calculated values were 0.84 (*p* < .001), 0.98 (*p* < .001) and 0.31 (*p* = .361), respectively. In the comparison of arterial and calculated pH values (Figure 2), one datapoint was visually and statistically identified as a major outlier. This sample pair was obtained from a patient with severe diabetic ketoacidosis. In this pair, the arterial pH was 7.07 and the calculated pH was 7.42, while the venous pH, from which the calculated value was derived, was 7.04.

## 4 DISCUSSION

To our knowledge, this is the first study focusing on the performance of the v‐TAC method in an ICU setting with critically ill patients with severe acid–base disturbances. Venous and calculated pH and PCO_2_ values showed similar congruency with arterial values in patients with alkalemia and moderate acidaemia. However, in patients with severe acidaemia, the v‐TAC method was unable to convert several venous values due to limitations of the method. Of note, a serious error of the method was detected as a very low venous pH value was converted to normal pH in a patient with severe metabolic acidosis.

Two studies have previously assessed the performance of the v‐TAC method with ICU patients and found agreement between calculated and arterial pH and PCO_2_ values superior to that between arterial and venous values.^19^, ^21^ None of the studies included patients with arterial pH values below 7.20, and the majority of patients in both studies had normal arterial pH values. In this study, calculated pH values were not superior, but reasonably alike venous pH values compared to arterial values, with the exception of patients with arterial pH values below 7.20. The cause of the flawed calculated pH value in the patient with severe metabolic acidosis is unknown but may be life‐threatening if clinicians blindly rely on the results. In the comparison of PCO_2_, the calculated values were slightly more alike arterial values than venous PCO_2_ values were. Neither venous nor calculated PO_2_ values resembled or correlated well with arterial values. In the patients with severe acidaemia, only three out of the venous PO_2_ values were converted to calculated values, of which only 1 was assigned a numeric value and the others the label ‘>10 kPa.’

The inaccuracy of the method at higher PO_2_ levels, and especially the limit of reported values above 10 kPa, renders the method unfeasible in certain patient groups. For instance, a PO_2_ target between 10 and 12 kPa is recommended in ICU patients with traumatic brain injury in Denmark.^25^ Wu et al. retrospectively assessed the outcome of both mechanically ventilated and spontaneously breathing patients with traumatic brain injury and PO_2_ values within 6 h of admission.^26^ They found that PO_2_ values below approximately 8 kPa were associated with poor outcomes compared to patients with PO_2_ values of approximately 8–13 kPa (odds ratio 5.3 95% confidence interval, CI 1.1–24.9, *p* = .034). Similarly, patients with PO_2_ values 8–13 kPa seem to have a better outcome compared to those with 13–26 kPa, although not statistically significant (odds ratio 0.466 95% CI 0.1–2.1, *p* = .325). Thus, the authors recommended PO_2_ targets of 8–13 kPa.^26^

Another limit of the method, the threshold of SpO_2_ of 75%, also has drawbacks in combination with the upper PO_2_ >10 kPa limit; the method may only be able to calculate actual numeric PO_2_ values within a small interval if samples are obtained from critically ill patients with both severe acidaemia and hypoxemia. This perspective is elaborated in the following: a decrease in pH will induce hb oxygen unloading, the Bohr effect and thereby increase PO_2_ levels, which is described as the well‐known right shift of the oxygen dissociation curve.^27^ Opdahl et al. obtained blood from human donors and produced oxygen dissociation curves by analysing the blood at different fixed pH and hb SO_2_ levels.^28^ By inspecting the produced oxygen dissociation curves a pH level of 7.1 and hb SO_2_ of 75% was equal to PO_2_ level of approximately 7 kPa, while a pH level of 6.8 and saturation of 75% were equal to PO_2_ of approximately 9 kPa.^28^ This means that in the patients with combined severe acidaemia and hypoxemia (e.g., pH levels just above 6.8 and SO_2_ near 75%), the v‐TAC method only produces numeric PO_2_ values in the range between approximately 9–10 kPa, which is impractical in clinical practice.

The basis for the SpO_2_ >75% limitation is not clear but may be related to inaccuracy of the peripheral pulse oximetry at lower SO_2_.^29^ The overestimation of calculated PO_2_ values at high arterial SO_2_ has previously been explained by the flat upper part of the oxygen dissociation curve, as only small changes in arterial hb SO_2_ result in major changes of arterial PO_2_.^14^ This may be the reason for the ‘>10 kPa’ label.

This study has several limitations. It was a single centre study conducted at an ICU receiving a broad heterogenous medical and surgical patient population. Therefore, transferability of results to other ICUs is limited. All sample pairs were collected as convenience samples, and if patients were severely unstable, lifesaving treatment did, naturally, overrule sampling from patients. Thus, the recruitment rate was rather low, and the most critically ill patients may not have been included. This introduces selection bias that may cause pitfalls on usage of the method in patients with the most severe acid–base disturbances to be missed. The low recruitment rate also resulted in a small sample size, which was a significant limitation of the study. While the v‐TAC method failed to calculate several values, the sample size may be too small to draw any conclusions about the value of the method in critically ill patients. The majority of studies assessing the performance of the v‐TAC method have used Bland and Altman statistics and plots to assess agreement between calculated and arterial values and venous and arterial values.^18^, ^19^, ^20^ This method is also recommended because correlation depends on the range and distribution of data, and it ignores any systematic bias between the variables measured.^30^ However, as a rule‐of‐thumb at least 100–200 independent measurements are required to avoid an unreasonable wide 95% CI of the limits‐of‐agreement to use the Bland and Altman plots for analysis.^31^, ^32^ Therefore, data from this study is primarily presented in a descriptive manner, and correlation plots are included to give a visual presentation of data.

In conclusion the venous and v‐TAC calculated pH and PCO_2_ values showed similar congruency compared to arterial values in patient with alkalemia and moderate acidaemia. In one patient with severe diabetic ketoacidosis, a severely abnormal venous pH value was converted to a normal calculated value. The limitations of the method caused an inability to calculate values or calculate numeric PO_2_ values. Therefore, the authors urge caution if the v‐TAC method is applied in patients suspected of severe metabolic acidaemia or in patients where accurate assessment of oxygenation is required until further research has elaborated on the limitations of the method. The sample size of this study may be too small to draw any conclusions about the value of the v‐TAC method in critically ill patients.

## AUTHOR CONTRIBUTIONS

PCDL, KAD, EFC and ML designed and conceptualised the study. ML, BSR and JBA analysed and interpreted the data. ML drafted the first version of the manuscript. BSR and JBA edited and commented on the manuscript. All authors contributed to the manuscript and read and approved the final version.

## CONFLICT OF INTEREST STATEMENT

The authors have no conflict of interest to declare.

## Data Availability

The data that support the findings of this study are available from the corresponding author upon reasonable request.
